# Efficient Synthesis for Altering Side Chain Length on Cannabinoid Molecules and Their Effects in Chemotherapy and Chemotherapeutic Induced Neuropathic Pain

**DOI:** 10.3390/biom12121869

**Published:** 2022-12-13

**Authors:** Wesley M. Raup-Konsavage, Diana E. Sepulveda, Daniel P. Morris, Shantu Amin, Kent E. Vrana, Nicholas M. Graziane, Dhimant Desai

**Affiliations:** 1 Department of Pharmacology, Penn State College of Medicine, Hershey, PA 17033, USA; 2 Department of Anesthesiology & Perioperative Medicine, Penn State College of Medicine, Hershey, PA 17033, USA

**Keywords:** cannabigerol, neuropathic pain, colorectal cancer, cannabinoid synthesis

## Abstract

(1) Background: Recently, a number of side chain length variants for tetrahydrocannabinol and cannabidiol have been identified in cannabis; however, the precursor to these molecules would be based upon cannabigerol (CBG). Because CBG, and its side chain variants, are rapidly converted to other cannabinoids in the plant, there are typically only small amounts in plant extracts, thus prohibiting investigations related to CBG and CBG variant therapeutic effects. (2) Methods: To overcome this, we developed an efficient synthesis of corresponding resorcinol fragments using the Wittig reaction which, under acid catalyzed coupling with geraniol, produced the desired side chain variants of CBG. These compounds were then tested in an animal model of chemotherapeutic-induced neuropathic pain and to reduce colorectal cancer cell viability. (3) Results: We found that all side-chain variants were similarly capable of reducing neuropathic pain in mice at a dose of 10 mg/kg. However, the molecules with shorter side chains (i.e., CBGV and CBGB) were better at reducing colorectal cancer cell viability. (4) Conclusions: The novel synthesis method developed here will be of utility for studying other side chain derivatives of minor cannabinoids such as cannabichromene, cannabinol, and cannabielsoin.

## 1. Introduction

In the United States, 150,000 new cases of colorectal cancer will be diagnosed in 2022 with an estimated 55,000 deaths caused by the disease [1]. Cisplatin, a commonly used chemotherapeutic agent, is used to treat colorectal cancer, albeit with negative side effects, including chemotherapeutic-induced peripheral neuropathy (CIPN), for which there is a lack of treatment options [2,3,4]. The prevalence of CIPN is agent-dependent, with reported rates varying from 19% to more than 85% [5] and is the highest in the case of platinum-based drugs (70–100%), including cisplatin [6]. This outcome results in an impaired quality of life for affected patients. Therefore, there is a need to identify alternative treatments for colorectal cancer, as well as identify anti-nociceptive agents capable of treating CIPN in patients undergoing cisplatin-based chemotherapy for colorectal cancer.

A number of cannabinoids have been found to reduce cancer cell growth with Δ^9^-tetrahydrocannabinol (THC) and cannabidiol (CBD)—the two most often studied cannabinoids for anti-cancer activity [7,8,9,10,11,12]. In addition to anti-cancer effects, evidence suggests that cannabinoids (e.g., cannabigerol (CBG), THC, and CBD) have anti-nociceptive properties, resulting in ongoing clinical investigations related to cannabinoid effects on pain [13,14,15,16,17,18]. The mechanisms by which cannabinoids reduce cancer cell growth as well as mitigate pain are currently under investigation. However, it is known that cannabinoids are promiscuous molecules with the ability to bind to a number of receptors involved in pain processing and inflammation (e.g., CB1, CB2, 5-HT_1A_, α2-adrenergic receptor) as well as induce immunogenic cell death [15,18,19,20].

Cannabis produces a number of bioactive compounds that are of pharmaceutical interest. Broadly, these molecules fall into one of three classes: terpenes, flavonoids, and cannabinoids (which are unique to the genus *Cannabis*). Over 100 different types of cannabinoids are produced by cannabis, with CBD and THC being the two most abundant and most well studied. The predominant cannabinoids in *Cannabis* cultivars have a 5-carbon side chain off of the aromatic ring; however, shortly after the discovery of the major 5-carbon phytocannabinoids, CBD and THC, two additional variations of these molecules were described with a shorter, 3-carbon, side chain, cannabidivarin (CBDV) and tetrahydrocannabivarin (THCV) [21]. More recently, CBD and THC variants with carbon side chains of 4, 6, and 7 carbons have been isolated from cannabis, albeit in much lower concentrations [22,23,24].

The biosynthesis of all cannabinoids begins with geranyl pyrophosphate and a benzoic acid, olivetolic acid in the case of the 5-carbon cannabinoids, by the enzyme geranyl-pyrophosphate-olivetolic acid geranyltransferase (GOT) [25]. This reaction produces CBG, and the respective variations, which serve as the precursor molecules to all other cannabinoids produced in the plant. However, in most cultivars, CBG is found at low levels because this precursor is efficiently converted to downstream products, which means that the variant molecules will therefore be present in trace amounts. Novel and trace side chain length variants of CBD and THC have previously been isolated from plant material [22]. Here, we set out to develop a novel synthetic mechanism to produce side-chain variants of CBG. Additionally, we tested CBG and CBG variants for potential anti-cancer activity in colorectal cancer cell lines, as well as anti-nociceptive properties in a model of chemotherapy-induced neuropathic pain (CIPN).

## 2. Materials and Methods

**Scheme 1:**
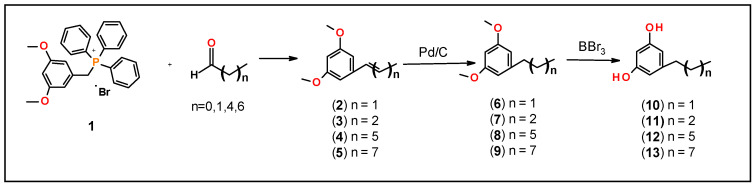
Synthesis of 5-alkyl substitute-1,3-dihydroxybenezene.

Thin-layer chromatography (TLC) was performed on aluminum-supported, precoated silica gel plates (EM Industries, Gibbstown, NJ, USA). Flash column chromatography was performed using silica gel SI 60. ^1^H NMR spectra were recorded on a 500 MHz Bruker mass spectrometer (Billerica, MA, USA). Proton chemical shifts are reported in parts per million (δ). The following abbreviations were used to designate chemical shift multiplicities: s = singlet, d = doublet, dd = double doublet, t = triplet, dt = doublet of triplet, m = multiplet. Mass spectrometry analysis was performed on a 4000 Q-trap hybrid triple quadrupole/linear ion trap instrument (Applied Biosystems/MDS Sciex, Waltham, MA, USA) at the proteomic facility of the Penn State College of Medicine, Hershey, PA. High Resolution Mass Spectrometry (HRMS) was performed on AB Sciex TripleTOF 5600 mass spectrometer (Farmington, MA, USA) with electrospray ionization (ESI) in positive-ion mode at the metabolomics core at Penn State University, University Park, PA. The sample was analyzed by flow infusion with a Prominence UFLC system (Shimadzu, Kyoto, Japan) at flow 300 μL/min rates of 0.1% formic acid in a mixture of methanol and water 60:40. MS1 and MS2 data were acquired using a declustering potential (DP) of 80 V. MS2 data were collected in IDA mode with collision energy (CE) of 40 V and collision energy spread (CES) 20 V. During the analysis, an ion spray voltage (IS) of 5500 V, curtain gas (CUR) of 35 psi, nebulizer gas (GS1) of 50 psi, heater gas 2 (GS2) of 55, and heater temperature of 550 C were applied. CBG was purchased from a commercial source (Cayman Chemical, Ann Arbor, MI, USA). 3,5-dimethoxybenzyltriphenylphosphonium bromide (**1**) was prepared as reported in the literature [26]. Briefly, 3,5-dimethoxybenzylbromide (10 g, 43.2 mmol) was heated under reflux with triphenyl phosphine (12.6 g, 47.6 mmol) in toluene (60 mL) for 6 h to give a quantitative yield of compound **1**. All starting materials were obtained from Aldrich Chemical Co. (Milwaukee, WI) and used without further purification. Synthesis of 5-alkyl substituted-1,3-dihydroxybenzene precursors was conducted as described below (and illustrated in Scheme 1).

**(E/Z) 1,3-Dimethoxy-5-(Prop-1-en-1-yl) benzene (2)**

3,5-dimethoxylbenzyltriphenylphosphonium bromide (**1**) (2 g, 4 mmol) in aqueous K_2_CO_3_ solution (0.1 M, 50 mL) and acetaldehyde (0.264 g, 0.337 mL, 6 mmol) were refluxed for 24 h, cooled to room temperature and cyclohexane (50 mL) was added and vigorously shaken for 30 min. The mixture was filtered, and the aqueous layer was extracted with cyclohexane (2 × 50 mL). Combined organic layers were dried over MgSO_4_, filtered, and evaporated. The crude olefin, **2** was purified on a silica gel column using Hexane: EtOAc (95:0.5) as an eluent to give mixture of olefins, **2** in 50% yield. ^1^H NMR (500 MHz, CDCl_3_): δ 6.50 (d, 2H, aromatic, J = 2.0 Hz), 6.39–6.30 (m, 1H), 6.28–6.11 (m, 2H), 3.80 (s, 6H, OCH_3_), and 1.87 (dd, 3H, CH_3_, J = 6.5 Hz).

**(E/Z) 1,3-Dimethoxy-5-(Butyl-1-en-1-yl) benzene (3)**

A mixture of olefins (E/Z)—**3** was prepared as described for olefin **2** using 3,5-dimethoxybenzyltriphenylphosphonium bromide (**1**) (4.0 g, 8 mmol) and propionaldehyde (0.69 g, 0.86 mL, 12 mmol) to give **3** in 91% yield. ^1^H NMR (500 MHz, CDCl_3_): δ 6.51 (d, 2H, aromatic, J = 2.5 Hz), 6.33 (d, 1H, aromatic, J = 2.5 Hz), 5.95–5.75 (m, 2H, CH), 3.80 (s, 6H, OCH_3_), 2.70–2.50 (m, 2H, CH_2_), 1.57–1.12 (m, 4H, CH_2_) and 1.12–1.00 (m, 3H, CH_3_).

**(E/*Z*) 1,3-Dimethoxy-5-(Heptyl-1-en-1-yl) benzene (4)**

Olefins (E/Z)—**4** was prepared as described for **2** using 3,5-dimethoxybenzyltriphenylphosphonium bromide (**1**) (4.0 g, 8 mmol) and hexanaldehyde (1.2 g, 1.48 mL, 12 mmol) to give **4** in 56% yield. ^1^H NMR (500 MHz, CDCl_3_): δ 6.41–6.20 (m, 2H, aromatic), 5.95–5.75 (m, 2H, CH), 3.97 (s, 6H, OCH_3_), 3.97 (s, 6H, OCH_3_), 2.32 (m, 2H, CH_2_), 1.46 (q, 2H, CH_2_, J = 7.5 Hz), 1.41–1.20 (m, 4H, CH_2_), and 0.96–0.88 (m, 3H, CH_3_).

**(E/*Z*)-1,3-Dimethoxy-5-(nonyl-1-en-1-yl) benzene (5)**

A mixture of olefins (E/Z)—**5** was prepared as described for **2** using 3,5-dimethoxybenzyltriphenylphosphonium bromide (**1**) (2.0 g, 4 mmol), and octanaldehyde (0.77 g, 0.94 mL, 6 mmol) to give olefin **5** in 95% yield. ^1^H NMR (500 MHz, CDCl_3_): δ 6.54 (d, 1H, J = 2.0 Hz) 6.47 (d, 1H, J = 2.0 Hz), 6.39–6.30 (m, 2H), 6.28–6.20 (m, 1H), 3.82 (s, 6H, OCH_3_), 2.40–2.32 (m, 1H), 2.25–2.20 (m, 1H),1.55–1.25 (m, 10H, CH_2_), and 0.92 (dd, 3H, CH_3_, J = 13.5 Hz and 6.5 Hz).

**1,3-Dimethoxy-5-propylbenzene (6)**

A mixture of **2** (1.00 g, 5.62 mmol) in EtOH (60 mL) and 10% Pd/C (100 mg) under 40psi of H_2_ atmosphere were shaken in Parr Hydrogenation apparatus for 24 h. The catalyst was removed by filtration over Celite and the solvent was evaporated under reduced pressure to give compound **6** in 88% yield; ^1^H NMR (500 MHz, CDCl_3_): δ 6.38 (d, 2H, aromatic, J= 2.0 Hz), 6.34 (t, aromatic, J = 2.0 Hz), 3.81 (s, 6H, OC*H*_3_), 2.57 (q, 2H, CH_2_, *J* = 7.5 Hz), 1.69–1.62 (m, 2H, CH_2_), and 0.97 (t, 3H, CH_3_,J_′_ = 7.5 Hz).

**1,3-Dimethoxy-5-butylbenzene (7)**

Compound **7** was prepared as described for compound **6** using a solution of **3** (576 mg, 3.0 mmol) in EtOH (30 mL) and 10% Pd/C (58 mg) to give **7** in 86% yield. ^1^H NMR (500 MHz, CDCl_3_): δ 6.39–6.38 (m, 2H, aromatic), 6.34 (bs,1H, aromatic), 3.81 (s, 6H, OCH_3_), 2.7–2.53 (m, 2H, Ph-CH_2_), 1.70–1.11 (m, 4H, CH2), and 0.97 (m, 3H, CH_3_).

**1,3-Dimethoxy-5-heptylbenzene (8)**

Compound **8** was prepared as described for compound **6** using a solution of **4** (702 mg, 3 mmol) in EtOH (50 mL) and 10% Pd/C (70 mg) to give **8** in 86% yield: ^1^H NMR (500 MHz, CDCl_3_): δ 6.38 (d, 2H, aromatic, J = 2.0 Hz), 6.34 (d, 1H, aromatic, J = 2.5 Hz), 3.81 (s, 6H, OC*H*_3_), 2.58 (t, 2H, CH2, *J* = 7.5 Hz), 1.64 (t, 2H, CH2, J = 7.5 Hz), 1.36–1.31 (m, 8H, CH2), and 0.92 (t, 3H, CH3, J = 7.5 Hz).

**1,3-Dimethoxy-5-nonylbenzene (9)**

Compound **9** was prepared as described for compound **6** using a solution of **5** (792 mg, 3.00 mmol) in EtOH (30 mL) and 10% Pd/C (83 mg) to give **9** in 93% yield. ^1^H NMR (500 MHz, CDCl_3_): δ 6.38 (d, 2H, aromatic, J = 2.0 Hz), 6.33 (t, 1H, aromatic, J = 2.0 Hz), 3.81 (s, 6H, OC*H*_3_), 2.58 (t, 2H, CH_2_, *J* = 8.0 Hz), 1.64 (m, 2H, CH_2_), 1.35–1.30 (m, 12H, CH_2_), and 0.92 (t, 3H, CH_3_, J = 7.0 Hz).

**1,3-Dihydroxy-5-propylbenzene (10)**

A stirring solution of **6** (200 mg, 1.11 mmol) in CH_2_Cl_2_ was cooled to −10 °C for 30 min. To this cold reaction solution, BBr_3_ (1 M solution in CH_2_Cl_2_, 2.78 mL, 2.78 mmol) was added dropwise. After the addition, the mixture was allowed to warm up to room temperature and stirred for an additional 24 h. The mixture was cooled to 0 °C and quenched with saturated NaHCO_3_ and stirred for an additional 30 min at room temperature. The aqueous layer was extracted with CH_2_Cl_2_ (2 × 25 mL), combined organic layers were dried over MgSO_4_, filtered, and evaporated. Purification on a silica gel column by using CH_2_Cl_2_:MeOH (98:2) as an eluent gave 79% yield of **10 [27]**. ^1^H NMR (500 MHz, CDCl_3_): δ 6.28 (d, *J* = 2.0 Hz, 2H, aromatic), 6.21 (S, 1H, aromatic), 2.47 (q, 2H, CH_2_, *J* = 9.0 Hz), 1.59 (q, 2H, CH_2_, J = 8.0 Hz), and 0.93 (t, 3H, CH_3_, J = 7.5 Hz).

**1,3-Dihydroxy-5-butylbenzene (11)**

Compound **11** was prepared as described for **10** using compound **7** (0.5 g, 2.58 mmol) and BBr_3_ (1 M solution in CH_2_Cl_2_, 5.16 mL, 5.16 mmol) to give compound **11** in 68% yield [28,29]. ^1^H NMR (500 MHz, CDCl_3_): δ 6.28 (s, 2H, aromatic), 6.20 (d, 1H, aromatic, J = 2.0 Hz), 4.89 (bs, 2H, OH), 2.60–2.40 (m, 2H, CH_2_), 1.63–1.10 (m, 4H, CH_2_), and 0.92 (t, 3H, CH_3_, J = 7.0 Hz).

**1,3-Dihydroxy-5-heptylbenzene (12)**

Compound **12** was prepared as described for **10** using compound **8** (0.98 g, 4.15 mmol) and BBr_3_ (1 M solution in CH_2_Cl_2_, 9.5 mL, 9.5 mmol) to give compound **12** in 91% yield [30]. ^1^H NMR (500 MHz, CDCl_3_): δ 6.27 (d, 2H, aromatic, J = 2.0 Hz), 6.20 (d, 1H, aromatic, J = 2.0 Hz), 2.51 (t, 2H, CH, *J* = 7.5 Hz), 1.59 (q, 2H, CH_2_, J = 7.5 Hz), 1.39–1.22 (m, 14H, CH_2_), and 0.91 (t, 3H, CH_3_, J = 6.5 Hz).

**1,3-Dihydroxy-5-nonylbenzene (13)**

Compound **13** was prepared as described for **10** using compound **9** (0.76 g, 2.88 mmol), and BBr_3_ (1 M solution in CH_2_Cl_2_, 5.8 mL, 5.8 mmol) to give compound **13** in 84% yield [31]. ^1^H NMR (500 MHz, CDCl_3_): δ 6.27 (d, 2H, aromatic, J = 2.0 Hz), 6.20 (bs, 1H, aromatic), 4.72 (bs, OH), 2.51 (t, 2H, CH_2_, *J* = 7.5 Hz), 1.61–1.58 (m, 2H, CH_2_), 1.33–1.28 (m, 12H, CH_2_), and 0.91 (t, 3H, CH_3_, J = 7.0 Hz).

**Scheme 2:**
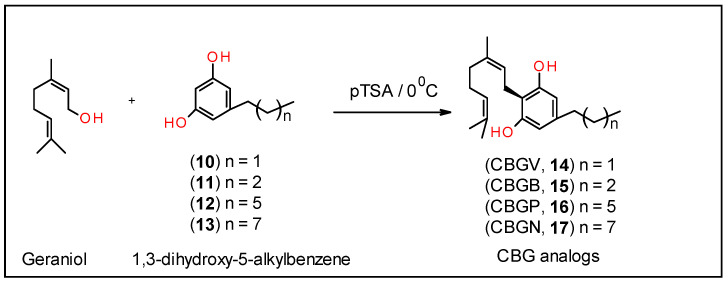
Synthesis of CBG analogs, **14**–**17**.

Syntheses of the CBG analogs of varying side chain lengths were conducted as described below and illustrated in Scheme 2.

**Cannabigerovarin (CBGV, 14):**

To a stirred cold solution of geraniol (203 mg,1.32 mmol) in anhydrous chloroform (20 mL) was added (over a period of 20 min) a solution of 1,3-dihydroxy-5-ethylbenzene (**10**) (131 mg, 0.73 mmol) and p-toluenesulfonic acid (14 mg) in anhydrous chloroform (15 mL) at 0 °C and under a positive pressure of nitrogen. After stirring in the same conditions for 14 h, the reaction was quenched with saturated of NaHCO_3_ (5 mL). The reaction mixture was extracted with EtOAc (3 × 15 mL) and the combined organic layers were washed with water, dried (MgSO_4_) and evaporated. The residue was purified on silica gel column by eluting with hexane:CH_2_Cl_2_ (1:1) to give 21% yield of CBGV (**14**) [32]. ^1^H NMR (500 MHz, CDCl_3_): δ 6.26 (s, 2H, aromatic), 5.33–5.28 (m, 1H, CH), 5.14 (bs, 2H, OH), 5.08 (t, 1H, CH, J = 7.0 Hz), 3.42 (d, 2H, Ph-CH_2_, J = 7.0 Hz), 2.56 (t, 2H, Ph-CH_2_, J = 7.5 Hz), 2.18–2.0 (m, 6H), 1.84 (s, 3H, CH_3_), 1.70 (S, 3H, CH_3_), 1.63 (s, 3H, CH_3_), 0.95 (t, 3H, CH_3_, J = 7.5 Hz); HRMS calculated for C_19_H_28_O_2_ + H: 289.2169; the observed value was 289.2091.

**Cannabigerobutol (CBGB, 15):**

Compound **15** was prepared as described for 1**4** using compound **11** (250 mg, 1.50 mmol) and geraniol (416 mg, 2.7 mmol) to give compound **15** in 18% yield. ^1^H NMR (500 MHz, CD_3_OD): δ 6.16 (d, 2H, aromatic), 5.26 (t, 1H, CH, J = 7.0 Hz), 5.09 (t, 1H, CH, J = 7.0 Hz), 3.27 (d, 2H, J = 7.5 Hz, Ph-CH_2_), 2.50–2.35 (m, 2H, Ph-CH_2_), 2.10–1.90 (m, 4H), 1.77 (s, 3H, CH_3_), 1.67 (S, 3H, CH_3_), 1.58 (s, 3H, CH_3_), 1.40–1.30 (m, 4H), 0.92 (t, 3H, CH_3_, J = 6.5 Hz); HRMS calculated for C_20_H_30_O_2_ + H: 303.2326; the observed value was 303.1941.

**Cannabigerophorbol (CBGP, 16):**

Compound **16** was prepared as described for **14** using compound **12** (208 mg, 1.0 mmol) and geraniol (278 mg, 1.8 mmol) to give compound **16** in 26% yield [33]. ^1^H NMR (500 MHz, CDCl_3_): δ 6.27 (S, 2H, aromatic), 5.30 (t, 1H, CH, J = 7.0 Hz), 5.08 (t, 1H, CH, J = 6.0 Hz), 5.02 (bs, 2H, OH), 3.42 (d, 2H, Ph-CH_2_, J = 7.0 Hz), 2.48 (t, 2H, Ph-CH_2_, J = 8.0 Hz), 2.19–2.10 (m, 4H), 1.84 (s, 3H, CH_3_), 1.70 (S, 3H, CH_3_), 1.62–1.57 (m and S, 5H, CH_2_ and CH_3_), 1.33–1.24 (m, 10H), 0.91 (t, 3H, CH_3_, J = 7.0 Hz); HRMS calculated for C_23_H_36_O_2_ + H: 344.2715; the observed value was 344.2657.

**Cannabigerononol (CBGN, 17):**

Compound **17** was prepared as described for **14** using compound **13** (307 mg, 1.5 mmol) and geraniol (417 mg, 2.7 mmol) to give compound **17** in 29% yield. ^1^H NMR (500 MHz, CDCl_3_): δ 6.27 (S, 2H, aromatic), 5.30 (t, 1H, CH, J = 7.0 Hz), 5.08 (t, 1H, CH, J = 6.5 Hz), 5.00 (bs, 2H, OH), 3.42 (d, 2H, Ph-CH_2_, J = 7.0 Hz), 2.48 (t, 2H, Ph-CH_2_, J = 7.5 Hz), 2.15–2.10 (m, 4H), 1.84 (s, 3H, CH_3_), 1.70 (S, 3H, CH_3_), 1.62 (s, 3H, CH_3_), 1.62–1.57 (m, 2H), 1.33–1.24 (m, 12H), 0.91 (t, 3H, CH_3_, J = 7.0 Hz); HRMS calculated for C_25_H_40_O_2_ + H: 373.3108; the observed value was 373.3089.

**Animals**

All experiments were performed in accordance with procedures approved by the Pennsylvania State University College of Medicine Institutional Animal Care and Use Committee (protocol 202001327). Wild-type C57BL/6 (The Jackson Laboratory, Bar Harbor, ME, USA) male mice (*n* = 35) were used for all experiments. Animals were grouped housed with a 12-h light/dark cycle with *ad libitum* food and water.

Cisplatin-induced peripheral neuropathy was induced as previously described [13,15,34]. Mice were injected with 4% sodium bicarbonate solution (1 mL, s.c.) administered just prior to cisplatin (5 mg/kg, i.p.) to prevent neurotoxicity and to minimize damage to renal function. Cisplatin was administered once a week for four weeks [15,35]. Mechanical allodynia was assessed before and after cisplatin treatment to confirm neuropathic pain state as described below.

Mechanical hypersensitivity was assessed using an electronic von Frey anesthesiometer (IITC Life Sciences Inc., Woodland Hills, CA, USA) with a semi-flex tip (IITC Life Sciences Inc., Woodland Hills, CA, USA), which was applied to the plantar surface of the right hind-paw with increasing force to prompt a paw withdrawal response. Mice were placed in one of eight small acrylic chambers placed on a wire mesh table (IITC Life Sciences Inc., Woodland, CA, USA), and acclimated to the chamber for 20 min before testing. The average of three tests were calculated with each test separated by at least 3 min.

To measure the effects of test compounds, 35 neuropathic male mice were randomly assigned to 7 groups (5 mice/group). During week 1, mice were injected with vehicle (DMSO, Tween 80, saline (1:1:18), i.p.), CBGV, CBGB, CBG, CBGP, CBGN, or the non-steroidal anti-inflammatory drug indomethacin (10 mg/kg, i.p., based upon our previous work with CBG [15]). Mice were then given a one-week washout period, and during week 2 were again randomly assigned to one of the 7 groups and injected with the test compounds. All von Frey experiments were performed by experimenters blinded to treatments.

**Cell lines**

The human CRC cell lines SW480, SW620, HT29, DLD-1, HCT115, LS174, and RKO were obtained from the American Type Culture Collection (ATCC, Manassas, VA) and cultured as previously described [7]. Briefly, cells were grown in Dulbecco’s Modified Eagle’s Media supplemented with 10% fetal bovine serum, 2 mM Glutamax, 10 U/mL penicillin, 10 µg/mL streptomycin, and 0.25 µg/mL Amphotericin B at 37 °C in 5% CO_2_. RKO cells were grown under the same conditions except that RPMI was used in place of Dulbecco’s Modified Eagle’s Media.

CRC cell lines were treated as previously described, except cells were seeded at a density of 10,000 cells/well [7] and 16 h later treated with vehicle (DMSO), CBGV, CBGB, CBG, CBGP, CBGN at 10 µM for 48 h. In all treatments, the DMSO was maintained at a constant 1%. Results in two cell lines (HCT116 and SW480) were confirmed by trypan blue staining. Cells were plated and treated as described above and after 48 h adherent and nonadherent cells were collected and stained with 0.2% trypan blue; cells were counted on a Countess 3 automated cell counter (ThermoFisher, Pittsburgh, PA, USA). For dose effect curves, cells were seeded as described above and then treated with Vehicle (DMSO, CBGV, CBGB, CBG, CBGP, or CBGN at concentrations of 333 nM, 1 µM, 3.3 µM, 10 µM, 18.56 µM, 33 µM, and 56µM. Cell viability for all experiments was measured using the MTT ((3-(4,5-dimethylthiazol-2-yl)-5-(3-carboxymethoxyphenyl)-2-(4-sulfophenyl)-2H-tetrazolium), Biovision; Milpitas, CA). MTT (0.5 mg/mL, 15µL) was added to each well and incubated for 2 h at 37 °C in 5% CO_2_. Formazan crystals were solubilized by adding stop solution (10% Triton X-100, 0.05% HCl in isopropanol) and vigorously pipetting the mixture. Absorbance was measured at 570 nm on a FlexStation 3 (Molecular Devices, San Jose, CA, USA). For each experiment, the cell line/treatment was measured from triplicate wells and the average was determined. Data are presented as the signal normalized to vehicle control.

**Statistics**

All results are shown as mean ± standard deviation. Statistical significance was determined using GraphPad Prism Software (9.3.1, San Diego, CA, USA) using a one-way ANOVA with Dunnett’s multiple comparison post hoc tests.

## 3. Results

First, to generate CBG and CBG variants, we synthesized corresponding resorcinol fragments. Several approaches have been reported in the literature for the synthesis of resorcinol derivatives [33,35]. We have developed an efficient method to generate the resorcinol fragments as shown in Scheme 1. This was accomplished in three steps involving: (1) preparation of corresponding olefins, **2**–**5** using 3,5-dimethoxybenzyl triphenylphosphonium bromide (**1**) and the corresponding aldehyde (Wittig reaction); (2) hydrogenation of the resultant E/Z-olefin mixture in Parr-hydrogenation apparatus to give compounds, **6**–**9**; and (3) deprotection of methoxy group of compounds, **6**–**9** by using BBr_3_ gave corresponding substituted resorcinol derivatives, **10**–**13**. All of these operations overall gave moderate yields (40–80%).

Each of these corresponding substituted resorcinol derivatives, **10**–**13**, were coupled with geraniol in the presence of catalytic amounts of *p*-toluenesulfonic acid monohydrate as shown in Scheme 2 to give the corresponding CBG analogs, **14**–**17**, in 20–30% yields [36]. The solubility of CBGV and CBG is between 25–50 mg/mL or around 80–180 mM in pure DMSO; the solubility drops by 100-fold or more when the compounds are in 25% DMSO [37].

**Figure 1:**
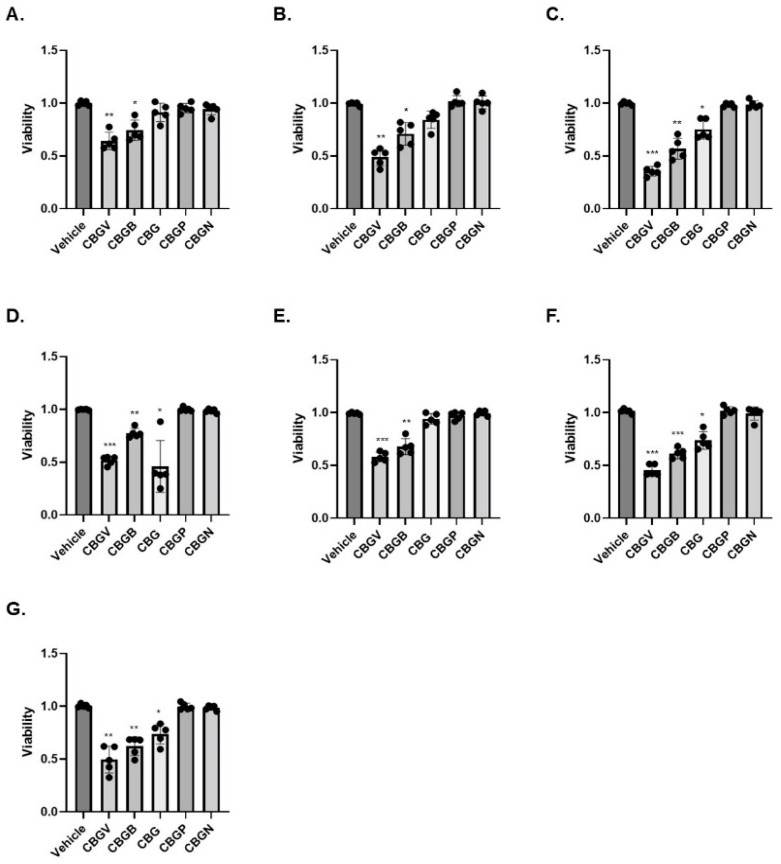
Effect of CBG side-chain variants on colorectal cancer cell viability. Colorectal cancer cell lines were treated with 10 µM of cannabinoid for 48 h and then viability was measured using the MTT assay. (**A**) SW480, (**B**) SW620, (**C**) HT29, (**D**) DLD-1, (**E**) HCT116, (**F**) LS174, and (**G**) RKO. *n* = 5 experiments per group. * *p* ≤ 0.05, ** *p* ≤ 0.01, *** *p* ≤ 0.005 vs. vehicle.

Given the potential of cannabinoids to evoke cancer cell death, we next investigated the effects of the CBG side-chain variants on cancer cell viability at 10 µM for 48 h. Following the 48-h timepoint, cell viability was assessed using the MTT assay. Our results show that CBGV, across all cell lines, demonstrated the greatest reduction in cell growth (Figure 1A–G). Additionally, we found that the 4-carbon variant, CBGB, decreased cancer cell viability, although not to the same extent as CBGV. Moreover, we found that the effects of CBG were dependent upon the cell lines tested, as CBG only reduced colorectal cancer cell viability in 4 of the cell lines tested (HT-29, DLD-1, LS174, and RKO, Figure 1C,D,F,G). Even in cell lines sensitive to CBG, CBGV remained more efficacious, except in DLD-1 cells where the CBG and CBGV had a similar effect on cell viability (Figure 1D). The longer 7 and 9 carbon variants (CBGP and CBGN) did not significantly influence cell viability in any of the cell lines tested. The results of the MTT assay were confirmed in HCT116 and SW480 cell lines by trypan blue staining (data not shown).

**Figure 2:**
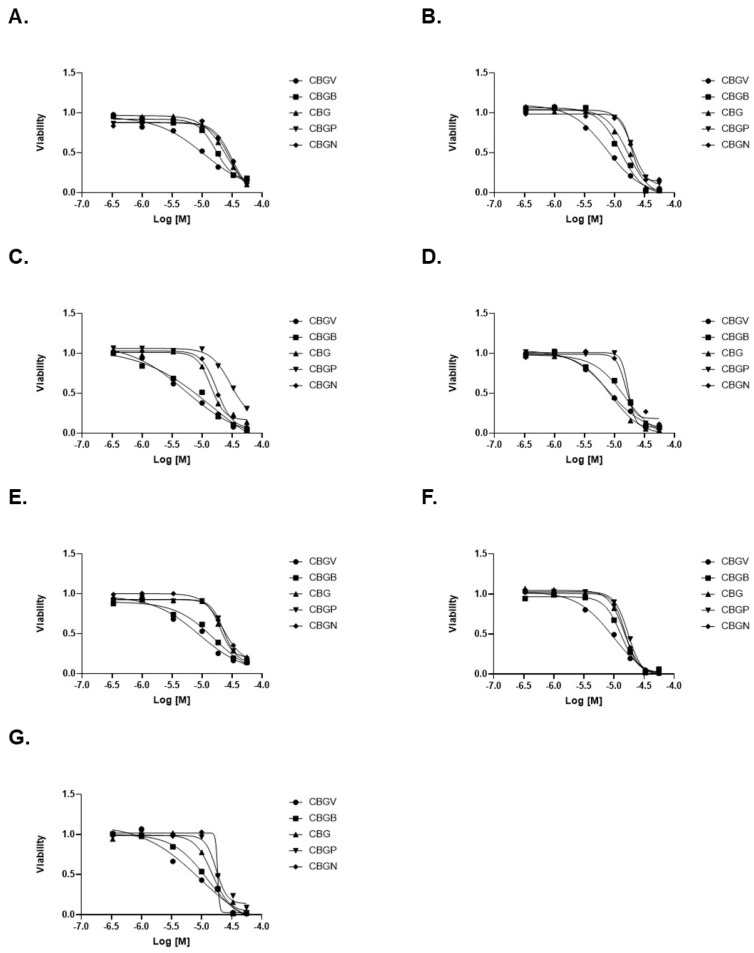
Dose response curves of CBG side-chain variants on colorectal cancer cell viability. Colorectal cancer cell lines were treated with varying doses of cannabinoid for 48 h and then viability was measured using the MTT assay. Representative dose response curves are shown for (**A**) SW480, (**B**) SW620, (**C**) HT29, (**D**) DLD-1, (**E**) HCT116, (**F**) LS174, and (**G**) RKO cell lines.

To better examine the impact of these compounds on colorectal cancer cell growth, we performed dose effect curves. Consistent with our data at 10 µM, we found that CBGV had the lowest IC_50_ value across all cell lines, except DLD-1 cells (Figure 2A–G, Table 1). We also found that only in DLD-1 cells was the IC_50_ value of CBG similar to that found with CBGV (Figure 2D, Table 1). The highest IC_50_ values were found for the molecules with the longer side chains (Figure 2, Table 1). In general, the IC_50_ values for CBGB and CBG were found to be between those observed for CBGV and the larger chain molecules (CBGP and CBGN) (Figure 2, Table 1).

**Figure 3:**
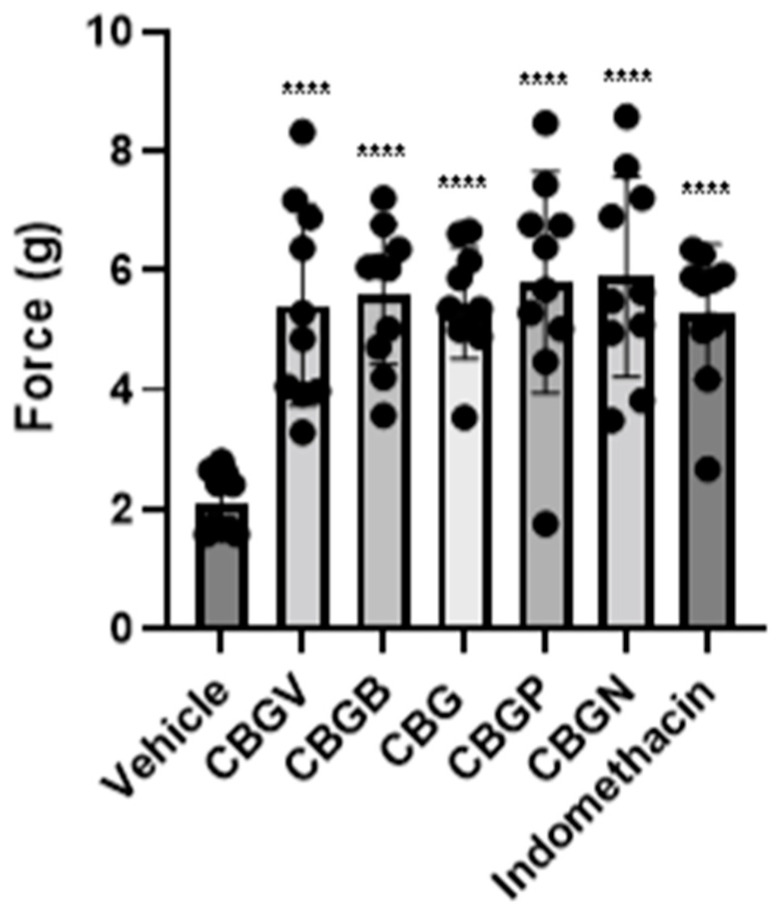
Effect of CBG side-chain variants on mechanical sensitivity. Neuropathic male mice were treated with 10 mg/kg of vehicle, side-chain variant of CBG, or indomethacin as a positive control and mechanical sensitivity was assessed using von Frey filaments. *n* = 10 mice per group, **** *p* ≤ 0.0001.

Next, we investigated the anti-nociceptive properties of CBG variants in a model of CIPN. Previously, we have demonstrated, using the von Frey test, that CBG (10 mg/kg i.p.) was effective at significantly reducing mechanical hypersensitivity in a preclinical model of CIPN [15]. Using this model, we investigated and compared the anti-nociceptive properties of the CBG side chain variants, CBGV, CBGB, CBG, CBGP, and CBGN. Neuropathic male mice were treated with vehicle control, a CBG variant, or the positive control indomethacin each at 10 mg/kg i.p. Mice then underwent von Frey testing of the hind-paw, 1 h following injections, to measure the force required to elicit a paw withdrawal response. Our results show that all variants were equally as effective as CBG and the positive control, indomethacin, in reversing CIPN (F_(6,63)_ = 9.56, *p* < 0.0001; one-way ANOVA with Tukey’s post-test). (Figure 3).

## 4. Discussion

Recently, a variant of CBD and THC has been identified in which the 5-carbon side chain is two carbons longer, these molecules were termed CBDP and THCP [22]. The authors went on to show that THCP binds to cannabinoid receptors with a higher affinity and was more effective at reducing pain than THC. In contrast, we did not see any impact of side-chain length on the ability of CBG to reduce pain in a mouse model of CIPN. However, we did find a significant anti-nociceptive effect of CBG and all CBG variants in a model of CIPN.

Our data indicate that side-chain length plays a role in the ability of CBG to reduce colorectal cancer cell growth in vitro. We found that, molecules with shorter side chains are more efficacious at reducing cell growth compared to longer side chains. Our findings with CBG are in contrast with previous work on CBDV and THCV which did not find a significant difference between these compounds and the more common 5-carbon variant (CBD and THC) [38,39,40]. Furthermore, neither the 4 or 7 carbon variants of CBD was found to have any greater impact on breast cancer cell growth than the 5-carbon molecule [41]. This could be due to the unique nature of CBG, which has been found to be an agonist of α2-adrenogeric receptors [42] and peroxisome proliferator-activated receptors (PPAR) α and γ [43,44,45,46], and the activation of these receptors has previously been reported to inhibit colorectal cancer cell growth.

It has been shown that side chain length can influence receptor binding for cannabinoids. For example, THCP has been shown to bind to CB1 receptors with a higher affinity than THC [22]. In contrast, THCV acts as an antagonist of the CB1 receptor, the opposite activity of THC, THCB, and THCP [24,47]. It is known that CBG binds with differing affinities and activities to a variety of receptors compared to THC and CBD [25]. One possible explanation for the differences we observe between the pain assay and cytotoxicity effects of these molecules is that different receptors mediate the analgesic and cytotoxic effects of CBG. Alternatively, the varying side chain lengths may create differing pharmacokinetics in vivo, thus normalizing their effects. Further studies on the binding of these novel molecules at known CBG receptors and additional studies on the mechanism that leads to cytotoxicity may provide useful insights into the mechanism by which CBGV and CBGB are slightly more cytotoxic but not more analgesic. These studies would also provide novel insights into how CBG interacts with known receptors.

## 5. Conclusions

We have developed a unique and adaptable process for generating cannabinoids with varying side chain lengths. Several recent reports have identified variants of CBD and THC with 3, 4, 6, and 7 carbon side chains; however, such side chain variants also likely exist for other cannabinoids such as cannabichromene (CBC) and must exist for CBG since this is the precursor molecule for the other cannabinoids. Surprisingly, we did not observe any effect of side-chain length regarding the ability to reduce neuropathic pain, which is in contrast to the data regarding THCP. However, we did find that CBG variants, such as CBG, produced significant anti-nociceptive effects in a murine model of CIPN. Importantly, we found that shorter side-chain variants of CBG were better able to reduce colorectal-cancer cell viability compared to longer-side chains.

## Figures and Tables

**Table 1:** IC_50_ values for CBG variants in Colorectal Cancer Cell Lines.

Cell Line	CBGV	CBGB	CBG	CBGP	CBGN
SW480	12.3 ± 2.5 **	15.5 ± 2.1 *	22.8 ± 3.4	23.9 ± 5.0	24.5 ± 6.0
SW620	8.1 ± 0.6 *	13.6 ± 1.7	16.2 ± 1.3	20.6 ± 3.6	23.4 ± 7.8
HT29	5.6 ± 3.3 **	12.5 ± 4.7	16.6 ± 3.3	25.2 ± 4.1 *	20.8 ± 3.6
DLD-1	8.3 ± 1.6	15.4 ± 1.4 **	7.9 ± 1.2	20.2 ± 3.9 ***	19.4 ± 3.7 ***
HCT116	9.3 ± 1.6 ****	15.8 ± 1.6 ***	21.6 ± 1.4	22.9 ± 0.1	22.3 ± 1.0
LS174	9.4 ± 1.1 ***	14.0 ± 3.1	16.7 ± 2.3	18.2 ± 1.1	17.3 ± 1.9
RKO	9.1 ± 2.9 **	13.1 ± 0.7	14.7 ± 0.5	17.5 ± 1.6	19.0 ± 1.6 *

IC_50_ values are from 3–4 independent dose effect curves and are presented as mean ± standard deviation, concentrations are in µM. * *p* ≤ 0.05, ** *p* ≤ 0.01, *** *p* ≤ 0.005, **** *p* ≤ 0.001 vs. CBG.

## Data Availability

Not applicable.
